# Breast cancer and the black swan

**DOI:** 10.3332/ecancer.2020.1050

**Published:** 2020-05-28

**Authors:** Michael Retsky, Romano Demicheli, William Hrushesky, Ted James, Rick Rogers, Michael Baum, Jayant S Vaidya, Osaro Erhabor, Patrice Forget

**Affiliations:** 1 Harvard T.H. Chan School of Public Health Boston, MA 02115-6021, USA; 2 University of Milan, Faculty of Medicine and Surgery, Milan 20133, Italy; 3 University of South Carolina, Columbia, SC 292012, USA; 4 Harvard Medical School, Beth Israel Deaconess Medical Center, Boston, MA 02215-5400, USA; 5 Emeritus Prof, University College London, London N19 5LW, UK; 6 University College London, London N19 5NF, UK; 7 Usmanu Danfodiyo University, Sokoto 840004, Nigeria; 8 University of Aberdeen, Aberdeen AB25 2ZD, UK

**Keywords:** breast cancer, bimodal relapse hazard, computer simulation, early relapse, surgery induced systemic inflammation, perioperative NSAID ketorolac, mechanisms, unmet need in Nigeria, proposed solution

## Abstract

Most current research in cancer is attempting to find ways of preventing patients from dying after metastatic relapse. Driven by data and analysis, this project is an approach to solve the problem upstream, i.e., to prevent relapse.

This project started with the unexpected observation of bimodal relapse patterns in breast and a number of other cancers. This was not explainable with the current cancer paradigm that has guided cancer therapy and early detection for many years. After much analysis using computer simulation and input from a number of medical specialties, we eventually came to the conclusion that the surgery to remove the primary tumour produced systemic inflammation for a week after surgery. This systemic inflammation apparently caused exits of cancer cells and micrometastases from dormant states and resulted in relapses in the first 3 years post-surgery.

It was determined in a retrospective study that the common inexpensive perioperative non-steroidal anti-inflammatory drug (NSAID) ketorolac could curtail the early relapse events after breast cancer surgery. A second retrospective study strongly confirmed this but an apparently underpowered prospective study showed no advantage.

We are analysing these data and are now proposing to test the perioperative NSAID at Beth Israel Deaconess Medical Centre with triple-negative breast cancer (TNBC) patients, the category that could respond best to the perioperative NSAID. If this works as well as we expect, we would then transfer this technology to low- and/or middle-incomes countries (LMICs), starting with Nigeria where early onset type of TNBC is common. There is an unmet need in LMICs, especially in countries like Nigeria (190 million population), for a means to prevent surgery induced relapse that we are attempting to resolve.

This work aims, thus, to describe eventual mechanisms, and ways to test a solution addressing an unmet need. But first, we consider the context, including within an historical perspective, important to explain how and why a Kuhnian paradigm shift may be considered.

## Part A—a contextual explanation: breast cancer data challenge the cancer therapy paradigm

### Introduction

This project started in 1993. Data were presented at a European conference by Romano Demicheli of the Milan National Cancer Institute. These data showed an unexpected bimodal relapse pattern for women treated with mastectomy only. At the same conference, Michael Baum described data from UK that also showed a bimodal relapse pattern.

### The black swan phenomenon or how anomalous data can generate a new model

From Wikipedia: *The phrase ‘black swan’ derives from a Latin expression; its oldest known occurrence is from the 2nd-century Roman poet ‘s characterisation of something being ‘rara avis in terris nigroque simillima cygno’ (‘a rare bird in the lands and very much like a black swan’). When the phrase was coined, the black swan was presumed not to exist. The importance of the metaphor lies in its analogy to the fragility of any system of thought. A set of conclusions is potentially undone once any of its fundamental postulates is disproved. In this case, the observation of a single black swan would be the undoing of the logic of any system of thought, as well as any reasoning that followed from that underlying logic.*

These data from Milan will be shown in full detail but as an overview, there were 1,173 patients treated by mastectomy that have been followed-up for over 10 years. More details are shown in Table 1. The persons in Italy are known to typically stay in the same town for generations and are generally considered to be compliant with physician directives. One person—Pinuccia Valagussa has been the data base manager since the project started. These are favourable factors for a high-quality database.

**Figure 1.**
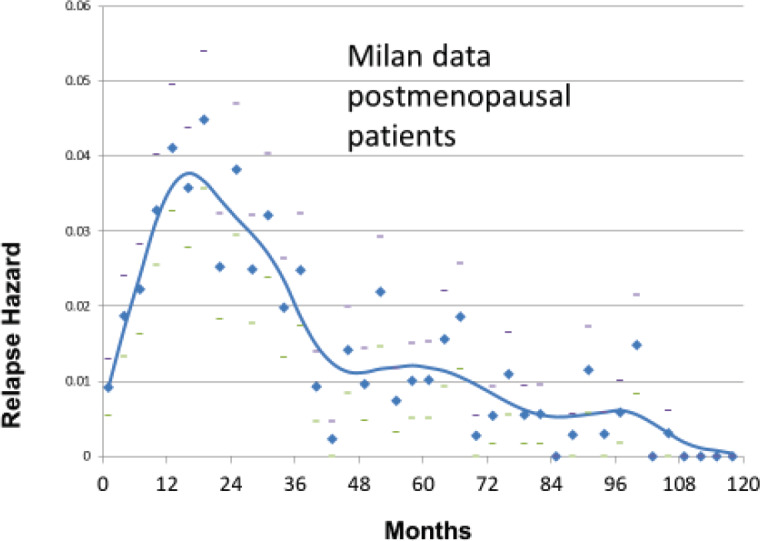
Relapse hazard for postmenopausal breast cancer patient vs. months since mastectomy.

**Figure 2.**
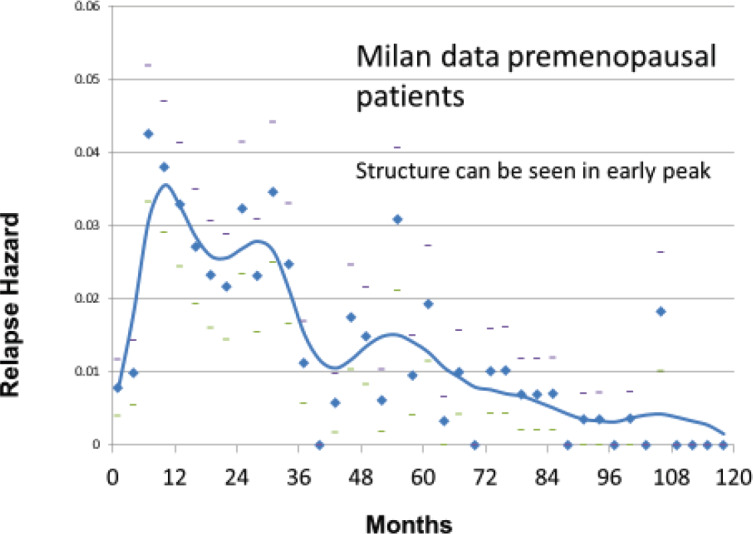
Relapse hazard for premenopausal breast cancer patients versus months since mastectomy.

**Figure 3.**
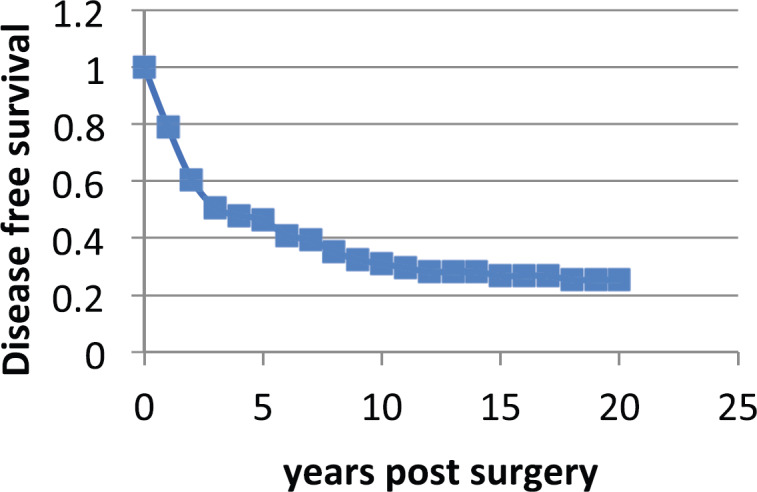
Disease Free Survival vs Years post-surgery from Bonadonna *et al* [1]. Data were transcribed from the original paper.

The Milan data are presented separately for the post-menopausal patients and the pre-menopausal patients in *hazard* or rate of relapse probability. These data are presented as Figures 1 and 2 and also in the more usual disease-free survival format as Figure 3 from Bonadonna *et al* [1]. Data were transcribed for Figure 3.

Retsky and Demicheli decided to collaborate. Their primary interest was to try and decipher the anomalous bimodal relapse hazard data. Demicheli checked and verified that these data were not an artefact resulting from specific periodic patient follow-up schedules that would artificially lead to such anomalies.

In a visit to Milan in 1994, Valagussa gave a computer disk with her data to Retsky. Demicheli then described what he thought to be the most basic sequence of the steps that a cancer must undergo from a single cell to a lethal size tumour.

Demicheli has MD, PhD degrees and did much experimental work on tumour growth. He described the simplest growth pattern that starts with a malignant cell that is in a non-dividing state. It can be dormant for a variable period of time. Once it starts to divide it can grow to a size of approximately 1 mm or 1 million cells. It cannot get larger than that until the host provides a blood supply or angiogenesis. After angiogenesis, the tumour can grow to be a detectable size and even achieve lethal size. This elementary growth model is considered valid now but 25 years ago in 1994 it would not have been so considered.

**Figure 4.**
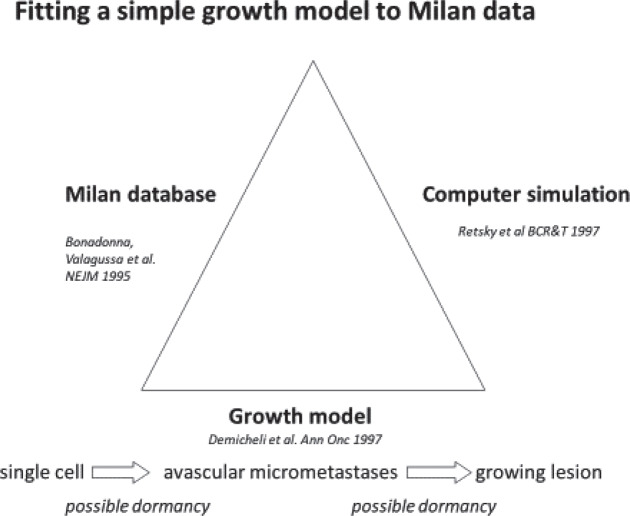
Description of the collaboration between Retsky, Demicheli and Valagussa.

Figure 4 describes the collaboration. Retsky [2] had done much work in computer simulation of tumour growth data starting in 1983 and undertook the task of using Demicheli’s model and Valagussa’s data to try and understand what the bimodal relapse peaks represented. Retsky had much good and bad experiences in working with breast cancer databases. He trusts the Milan data and much of that was due to the presence of Pinuccia Valagussa. Retsky got a small grant from NIH to make a computer simulation of breast cancer using the Milan data.

A main tool that was developed in this research project was a computer simulation of tumour growth from single cells to relapse size tumours. Our concept was to build a computer simulation of tumour growth based as much as possible on published human data. A major resource was a Retsky *et al* paper [3] showing published data for over 100 untreated spontaneous tumours mostly in humans.

Among the data in that 1990 paper, there was one report demonstrating dormancy followed by regrowth for an untreated primary breast tumour in a 78-year-old woman [3–5]. Her husband had died recently after spending their life savings in futile treatment of cancer of the larynx. She stubbornly refused to submit to surgery but allowed the physician to take occasional roentgenograms. There were four X-rays taken over 22 months. We have seen no other untreated breast cancer data in the literature with three or more measurements taken over more than 1 year. The tumour was detected with a cross-sectional area of 4.5 sq. cm. essentially in a growth plateau (7,200–day doubling time) for almost a year and then it began to grow (180–day doubling time) before it was removed and examined after another year. It was unremarkable adenocarcinoma. A 4.5 sq. cm. tumour with a constant doubling time of 7,200 days leads to the impossible result that the tumour started growing 655 years earlier. The tumour must have grown and then gone dormant prior to diagnosis.

The first version of the simulation was based on the growth model described in Figure 4. It turned out to be well suited to simulate the relapses from years 4 to 10 for the Milan data shown in Figures 1 and 2. However, it was unable to simulate the early relapses in years 1–3. The early relapse peaks were too sharp for that original model.

We were also aware of human data showing sudden growth of pulmonary metastases after amputation of osteosarcoma in a young boy in a 1968 paper by Smithers [3,6]. Thus, it was reasonable to include possible surgery induced exits from dormancy. With that addition, the computer model was well able to simulate all relapses in years 1–10 of Figures 1 and 2.

Based on the simulation that was fit to the Milan data, we hypothesised that the relapses at 10 months were surgery induced angiogenesis of avascular micrometastases. The relapses at 30 months were surgery induced single cell division followed by stochastic angiogenesis. The relapses that had a shallow peak at 60–70 months with a long tail extending to 10 and more years were not stimulated by surgery. The differences between premenopausal and postmenopausal patients are described in Table 1.

There were few relapses at 10 months for postmenopausal patients so the 10-month peak and the 30-month peak were indistinguishable in the data, thus appearing as a single 18-month peak.

**Figure 5.**
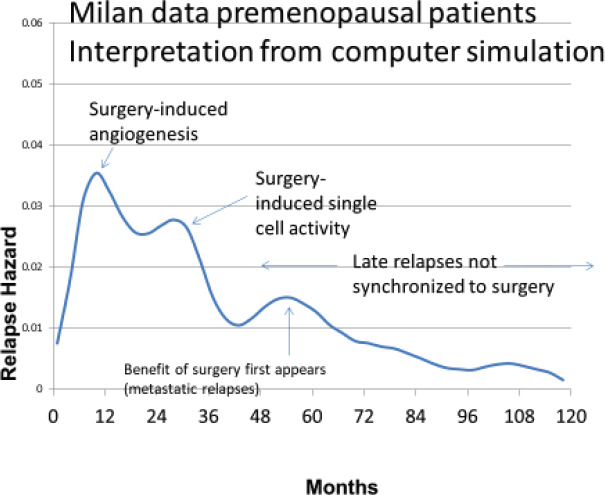
Simulation of breast cancer using the Milan data.

The results of the simulation are shown in Figure 5 superimposed on the relapse hazard for the premenopausal patients.

According to the simulation, the relapses in the first 3 years after surgery are iatrogenic or precipitated somehow by an intervention at or near the time of surgery. The relapses at the 10th month are dormant avascular micrometastases that were induced somehow into angiogenesis by the surgery and appeared as detectable metastases at 10 months. The 30-month events were single dormant cells that were induced into division by the surgery and then underwent angiogenesis stochastically and showed up as relapses at approximately 30 months.

The small shallow peak at 60 or 70 months represents the more or less natural history of breast cancer. The point at which the benefit of removal of the primary tumour to reduce metastases first appears at about 60 months. The peaks at 60–70 months and 100 months are currently under investigation. The quantitative results are listed in Table 1.

Ironically, Retsky was treated for Stage IIIc colon cancer during 1995–1997 while doing the computer simulation. This became the first use of metronomic chemotherapy for early stage cancer and was documented by Bernstein in Propublica [7]. The therapy was designed by W. Hrushesky.

Retsky and Demicheli later edited a book on this project that was published by Springer-Nature in July 2017 [5]. Much of the information presented in this paper was published in the book. Since the book is available online at Retsky’s Harvard website (https://www.researchgate.net/publication/321146437_Perioperative_inflammation_as_triggering_origin_of_metastasis_development), we can go over some of this information in an abbreviated fashion.

### How this new model can challenge the current paradigm

The Foreword to the book was written by Robert Weinberg of MIT. There are two provocative comments at the end of Weinberg’s Foreword that need to be presented here.

He notes that if demonstrated definitively, the findings in this book lead to a notion that is likely to be accepted only reluctantly by many in the clinical oncology community: primary tumour resection does not provide an undiluted benefit to the breast cancer patient in terms of long-term survival. He also notes that the findings reported in the book are likely to profoundly influence future surgical oncology and post-surgical treatment protocols.

A clear bimodal relapse pattern can be identified in figure from a 1984 paper by Fisher *et al* [8]. This figure is transcribed from the original. The original Fisher, Sass and Fisher figure can be seen in the Springer-Nature book on page 24. Likewise, the original Bonadonna, Valagussa NEJM 1995 figure can be seen on page 23.

These are disease free survival data for patients treated only with mastectomy and grouped by the number of axillary nodes positive. The number of nodes positive is well known to be the most important prognostic factor for relapse in breast cancer. The curve on top is for patients with zero nodes and the curve on the bottom is for patients with more than 12 nodes positive for cancer.

Consider the zero node patients. Mastectomy alone will cure about 80% of these patients. These 80% will never have a relapse. Of the 20% that relapse, 10% or half of the relapses occur in the first 3 years and then there is a period from 3 years to 5 or 6 years with few relapses and then the remaining 10% of relapses occur between 6 and 10 years.

Now looking at the patients with more than 12 nodes positive, essentially all the patients relapsed. The prognosis for >12 nodes was very poor, especially prior to the time of adjuvant chemotherapy in the 1980s. Of the essentially 100% that relapse, about 85% relapse in the first 3 years and then there are few relapses until 6 years when the remaining 15% relapse.

It is very interesting that while the magnitudes differ greatly, the timing of the early and late relapses for *N* = 0 patients is virtually the same as for the >12 node patients. This suggested to us that something happens to about 10% of patients with zero nodes that causes them to relapse within 3 years of surgery. Something similar seems to occur to 85% of patients with >12 nodes that causes them to relapse within 3 years of surgery. That apparently is the major difference between the zero node and the >12 node populations.

This finding may not be such a major surprise to the medical community. National Institutes of Health has long denied that surgery can cause cancer to spread ‘The chance that surgery will cause cancer to spread to other parts of the body is extremely low’ [9]. However, as noted by Komaroff in NEJM June 14, 2018, ‘Clinical lore says that resection of a primary tumour can cause previously inapparent metastatic deposits to flare’ [10]. Therefore, overlooking any mendacity, the possibility that primary surgery can cause metastatic activity may not be as revolutionary as we originally thought.

**Figure 6.**
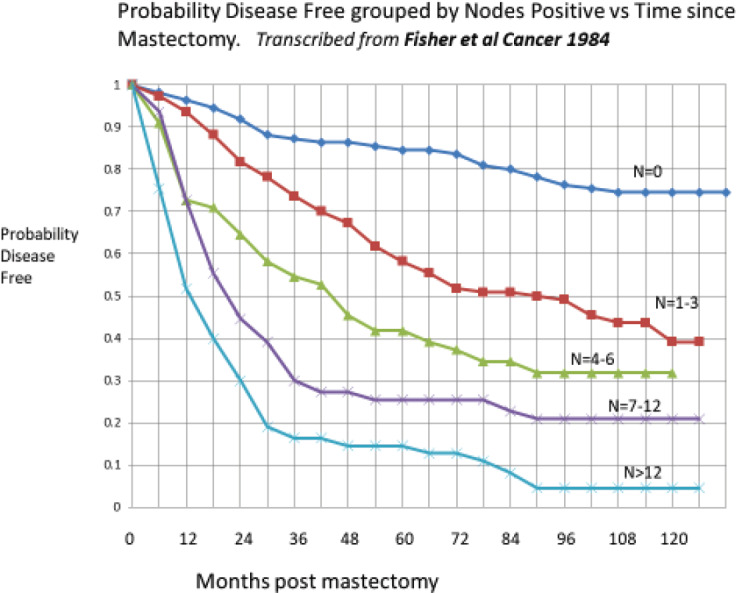
Data from Fisher, Sass and Fisher [8]. This figure has been transcribed from the original. The relapses in the first 3 years and after 6 years can be seen especially for the *N* = 0 and the *N* > 12 data. The magnitudes vary but the timing is quite similar from *N* = 0 to *N* > 12 data.

Taking a clue from the computer simulation shown in Figure 5 and the Fisher Sass and Fisher data in Figure 6, there appears to be a connection between the number of nodes positive and the probability of surgery-initiated relapses within 3 years of surgery.

Figure 6 has relevance to the early detection of breast cancer. Mammography was initiated to detect cancer in a most curable state—early in the disease and thus mostly with zero or few nodes positive and small tumours. It is clear from Figure 6 that such patients are easiest to cure with surgery only. Adjuvant therapy would provide additional benefit. Patients diagnosed with many nodes positive are considered a failure of early detection. Numerically, with mammography the percentage of patients age 40–49 diagnosed with positive nodes is 22.4% while without mammography the percentage is 43.4% [11]. *The speculative question arises: Would early detection be nearly as important if the surgery-induced relapses can somehow be prevented?*

After becoming familiar with relapse hazard and DFS data in breast cancer, reasonably similar patterns can be identified in other cancers (Table 2).

### Was this problem known 2,000 years ago?

A paper by Baum [29] described his comment about the bimodal relapse pattern. In that same book, a paper by Irving Ariel [30] provides a deep historical perspective of the treatment of breast cancer. It was most interesting in that Ariel presented information written by Galen of Pergamum and Aulus Cornelius Celsus. These were two famous historic Greek and Roman scientists and physicians. Their statements are shown in Table 3. These statements have been translated several times so the exact wording may not be perfect.

We are apparently latecomers to a 2000 year old problem in oncology.

Cacotheses is a term frequently used up to the 1800s and refers to small but apparent tumours. According to the theory presented in this document, primary surgery causes metastatic activity from dormancy and adjuvant chemotherapy is administered to partially offset this metastatic activity. If we could prevent metastatic activity from becoming stimulated after primary surgery, would adjuvant chemotherapy be needed and would it be effective? Theoretically the answer is no to both questions. Further, according to data from Agresti *et al* [31], Her2 + patients have significant surgery induced exit from dormancy. What might that mean? These are very serious issues and need to be examined in careful detail before any clinical changes are considered.

### Can this explain important clinical observations in breast cancer?

These surgery-induced effects are so large that they should be observable in clinical data if we knew what to look for. Following are four such effects that can be explained by our theory.

#### Adjuvant chemotherapy

Adjuvant chemotherapy works particularly well for premenopausal N+ patients. The curative effect of adjuvant chemotherapy is mostly confined to premenopausal node positive patients in which case approximately 12% are cured. For other categories, the curative rate is in the lower single digits [32]. This is reasonable since surgery induced angiogenesis produces the most rapidly growing cancer deposits possible just post-surgery when it was empirically found that maximum tolerated chemotherapy works best [33].

Notice that the coauthors in this 2004 paper [33] include Gianna Bonadonna with 30 years experience in developing adjuvant chemotherapy protocols and Judah Folkman with 30 years experience in developing the science that a tumour needs angiogenesis in order to grow larger than a mm or so.

#### Mammography screening

Mammography works better for women age 50–59 than age 40–49 years.

Mammography researchers knew that the early detection would be beneficial but they did not know the quantitative benefit so they conducted clinical trials in US, UK and Sweden. When examining the results of early detection for women age 50–59, they found 20% survival advantage that appeared early and was similar across the various trials. However, when they looked at trials for women age 40–49, there was an anomaly. Early in all trials (US, UK and Swedish overview), there was excess mortality for the intervention arm compared to controls. This caused great confusion and consternation and precipitated what came to be known as the mammography wars [34–36].

We could explain this. For women age 40–49 years, who are mostly premenopausal, there are some women who have breast cancer at detectable size and some of these who also have avascular microdeposits. When these cancers are detected, they are surgically removed which activates the dormant metastatic deposits. These appear as detectable relapses at about 10 months post-surgery. Since survival after relapse is approximately 2 years, if this is true, there would be a surge in mortality starting 3 years after the screening trial begins. That is precisely what was found in US, UK and Swedish overview data [37]. The magnitude was also calculated from the Milan data and agreed with mammography trial data. We published several papers explaining this.

Before publication, the 2005 paper [37] happened to become known to Amy Marcus, a Pulitzer Prize winning reporter at Wall Street Journal, since she was a guest at several of the Folkman lab meetings. On the day the paper came out (September 13, 2005), Harvard issued a press release and Amy Marcus published a 1,200-word report in the Wall Street Journal [38]. This drew much attention and we received a number of letters from readers. One in particular from Dr. Isaac Gukas led us into a new direction of our research.

Dr. Gukas was a surgeon in UK. He was originally from Nigeria where he practiced oncology for about 15 years. He wrote in his letter that our paper could explain what happens in Nigeria. Breast cancer in Nigeria is typically detectable as a lump at age mid 40 years. Relapses occur very soon after mastectomy and this became well known among the population. The word Gukas used to describe this effect was that the surgery ‘provoked’ the cancer. As a result, women who find breast lumps would usually see an herbalist rather than a surgeon and then go back to their village where they eventually die from untreated breast cancer.

Baum, Gukas and Retsky met at the Royal Medical Society in London in 2006. Gukas described a time in Nigeria when he had a televised debate with an herbalist on how to best treat breast cancer.

#### Racial disparity in outcome (inversion at age 57 years)

In the US, there is 1.5 excess mortality from breast cancer for African Americans (AA) compared to European Americans (EA) Retsky *et al* [39]. However, there is an inversion at age 57 years [40]. AA who are diagnosed under age 57 years have excess mortality compared to EA but if diagnosed over age 57 years have superior mortality outcome compared to EA. This means that we cannot explain the excess mortality of AA on reduced access to quality medical care. It must be biological. This was considered good news since it is far easier to fix a biological problem than to fix a socio-economic problem. Demicheli *et al* [41] is particularly important.

#### ‘Aggressiveness’ of breast cancer in young women

Breast cancer is often referred to by clinicians as aggressive in young women. From the clinicians’ viewpoint, premenopausal breast cancer appears as aggressive since young patients often relapse soon after primary surgery, while from our perspective, it is clockwork relapse at 10 months from surgery.

At this point, it was apparent that something happens at or about the time of surgery to initiate exit from dormancy and result in relapses within 3 years of surgery. These relapses within 3 years of surgery comprise 50% to 80% of all relapses. (Reminder that we are still speaking of breast cancer data from patients treated before the routine use of adjuvant chemotherapy and hormone therapy.) We did not know what that process was or how to stop it. However, it was clear that something had to be done before or at the time of surgery.

## Part B—mechanisms, testing a solution and addressing an unmet need

### Two events occurred that led us to consider a mechanism based on inflammation as a driving force for metastatic activity. Could inflammatory processes be therapeutic targets?

#### El Saghir *et al* paper 2005 [42]

First, a paper was submitted by El Saghir *et al* to BMC Cancer and Retsky was asked to review it. This was a case report of a 50-year-old Lebanese male who was diagnosed with inoperable non-small-cell-lung-cancer. The patient was treated with radiation and released from the hospital. His prognosis was very poor but at least for a while he could lead a normal life. While driving his car, he bumped his head on the sun visor and within 30 days a 7 cm tumour grew there. El Saghir suggested this could be an example of surgery-induced growth that Retsky and Demicheli had been describing.

Having a computer simulation allowed us to numerically consider if the El Saghir *et al* patient’s tumour growth could be what we have been writing about. It was clearly numerically not possible. But then we did not know what could have caused this sudden growth. Taturo Udagawa suggested that we look at a paper from Mina Bissel’s group on inflammation seen in an Avian Roux Sarcoma model. The paper was Martins-Greens *et al* [43]. Also, see Dolberg 1985 [44]. The inflammation is responsible for the development of wound-induced tumours in chickens infected with Rous sarcoma virus and described a virus model in which tumour would grow at any point where a wound was produced but the interesting part was that this was controllable with inflammation. If inflammation was allowed, the tumour would grow but if inflammation was prevented, no tumour would grow at the wound site.

Retsky’s published commentary to the El Saghir *et al* [45] paper stated: ‘The unusual isolated and exaggerated situation allowed El Saghir *et al* to observe what may be a new and possibly important hematologic pathway: inflammation as a facilitating precursor to tumour’. However, a virus particle is 1/100 of the size of a cancer cell so the mechanism reported in the Martins-Green paper was not a clear link to explain the El Saghir report but we were alerted to the possible importance of inflammation as a facilitating mechanism for wounding leading to cancer activity. http://www.biomedcentral.com/1471-2407/5/94/comments

#### Forget *et al* paper 2010 [46]

**Figure 7.**
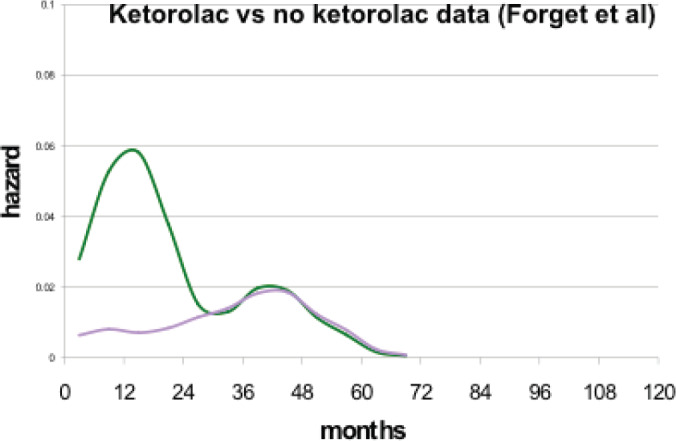
Breast cancer relapses after breast cancer surgery, observed by Forget *et al* in 2010. Purple is for patients having received ketorolac vs. no ketorolac in blue.

Second and much more important, a paper [46] was published by a Brussels anaesthesiology group that reported perioperative use of non-steroidal anti-inflammatory drug (NSAID) ketorolac significantly reduced early relapses in a retrospective study. This group is based at a teaching hospital so they need to expose the anaesthesiology residents to all the drugs used in anaesthesia. There were six anaesthesia drugs considered. There are now three NSAIDs that are available as iv but just one—ketorolac—was significantly used in this centre at that time. Their data are shown as Figure 7. There was a clear strong reduction in relapses for perioperative ketorolac in a retrospective study of 320 consecutive breast cancer patients given mastectomy by one surgeon and treated with conventional adjuvant therapy.

**Figure 8.**
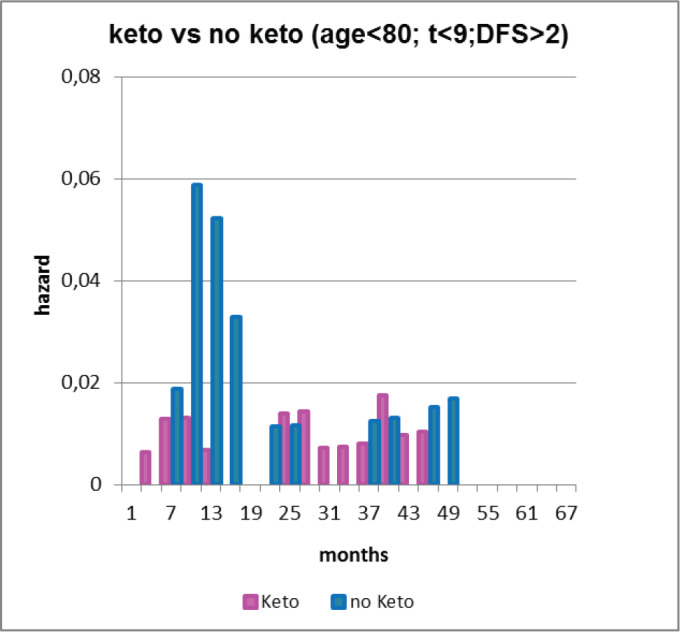
Forget *et al* data updated by Sarah Amar and analyzed by Demicheli. Note the five-fold reduction in relapses months 9–18 (3 versus 15 events). This histogram is useful to visually show the large reduction in early relapses.

After a visit to Brussels by Demicheli and Retsky, data were updated by Sarah Amar and analysed by Demicheli. This is shown in Figure 8.

Mathematically, in chaos theory a minute change in a complex system can have large effects elsewhere. Perhaps cancer can be considered a system in chaos [47].

**Figure 9.**
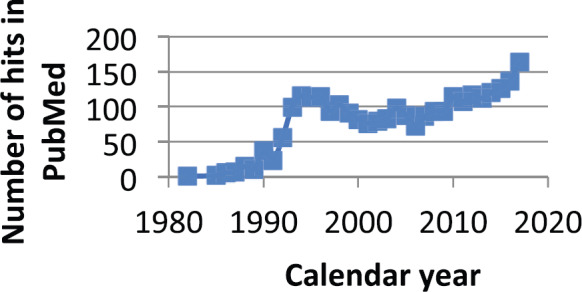
Number of times ketorolac is mentioned in PubMed since 1980. It was apparently brought into significant use starting in 1990.

Considering Figure 9, it may be a coincidence but there has been a steady small reduction in breast cancer mortality starting in 1990. It has been claimed that this is due to improvements in early detection and also in effect of therapy. Is it also possible that the use of ketorolac is another reason?

### What mechanisms could explain these data and our computations?

We naturally suspected something to do with inflammation. At this point, we undertook the task of looking for correlations among the fields of surgery, dormancy, inflammation, immunology, oncology, circulating tumour cells and wounding. We of course knew these were very large and complex fields (Table 4).

#### Platelets

Platelets actively sequester angioactive factors and degranulate in the presence of inflammation [62]. Platelet count drops by about 10% and VEGF increases also by 10% for a week after surgery. This could account for the relapses 10 months after surgery that happen for all categories of patients but most pronounced for premenopausal node positive patients.

#### Neutrophils

Neutrophil extracellular traps can capture circulating cancer cells [52, 53] and since there is increased capillary permeability after surgery [67] and neutrophils have the general ability to extravasate, it is possible for this to cause sudden increase in CTCs to become trapped in distant organs within a week of surgery and cause relapses 30 months later.

The Bonnelykke—Behrndtz Chapter in the Springer book [69] describes an experiment in zebrafish where neutrophils are attracted to a wound. That is reasonable since the main function of neutrophils is to aid in wound healing. However, the neutrophils are diverted to nearby preneoplastic cells that then start to divide. Zebrafish are semitransparent and neutrophils can be colour labelled allowing visual tracking of their paths. Fish are a far older species than humans but this could be a mechanism to explain 30 month relapse. That it remains a possibility is also addressed by Dillekas and Straum [65].

#### Krall *et al* protumoural immunological balance (2018)

In a major project at MIT in Dr. Weinberg’s lab, Krall *et al* [70] developed an animal model for dormancy of single cancer cells. In mice with an intact immune system Krall *et al* were able to inject cancer cells that became dormant and stayed viable. If the immune system was compromised this did not happen. Any surgery would cause activated monocytes from the marrow to travel to the sites of the pseudo-metastases and become tumour-associated macrophages and suppress the immune system locally causing exit from dormancy.

From our particular perspective this was very important since they could do an experiment. We are skilled in analyzing clinical data but cannot do an experiment. Krall *et al* were able to show that they could operate on the left side of a mouse and tumour grew on the right side (and vice versa). This process could be controlled with a perioperative NSAID. MIT issued a press release that received national attention.

#### Panigraphy *et al* (2019)

Another very important paper by Panigrahy *et al* [84] reports in animal models that Ketorolac and resolvins exhibited synergistic antitumour activity and prevented surgery or chemotherapy-induced dormancy escape.

#### Are there specific clinical data that support the hypothesis that post-op inflammation provokes metastases?

Reference to data from Kita *et al* [85]—Does Postoperative Serum Interleukin-6 (IL-6) Influence Early Recurrence after Curative Pulmonary Resection of Lung Cancer?

Kita *et al* [85] examined the influence of inflammatory cytokine levels on postoperative early recurrence in patients who underwent curative lung cancer surgery. In 107 patients who underwent curative pulmonary resections for non-small cell lung cancer from November 2007 to June 2008, they measured IL-6 levels preoperatively, and on postoperative day (POD) 0, 1, and 2.

One year after the date of enrolment of the last patient, they investigated survival status of each patient and identified a group with recurrence.

Among the 107 patients, 29 patients developed recurrence with a mean follow-up of 18.1 months (range 14 to 21). Clinical stage was significantly more advanced in the recurrence group than in the non-recurrence group (*p* = 0.005). Serum IL-6 levels on POD 1 were significantly higher in the recurrence group than in the non-recurrence group (*p* = 0.007). Stage and serum IL-6 levels on POD 1 were significant independent predicting factors for postoperative early recurrence (*p* = 0.006, *p* = 0.003). They concluded that the higher the serum IL-6 levels on POD 1, the higher the risk of early postoperative recurrence.

On the right is presented the usually accepted relapse mechanism. Cancer cells leave the primary and get deposited in reservoirs where they can reside for variable times up to a number of years. Eventually, some escape and ultimately lead to relapses. On the left is our proposal. As a result of the primary surgery, there is period lasting a week during which systemic inflammation exists and this can cause increased capillary permeability leading to circulating cancer cells getting trapped or deposited in distant organs. Also, platelets degranulate releasing VEGF causing angiogenesis of dormant avascular deposits. Resulting relapses in the first three years are a result. We do not think cancer cells are often released during surgery.

#### Are there other clinical data to show that ketorolac prevents early relapses?

In addition to the initial Forget *et al* [46] report, there are retrospective data for breast (Desmedt *et al* [79]) and for ovarian (Hudson *et al* [86]). As reported by Hudson:

‘At the 60‐month follow‐up, 3/17 ketorolac‐treated (18%) and 40/92 non‐ treated patients (43%) had died of ovarian cancer (log‐rank test *p*‐value = 0.09). Stratified log‐rank tests for categorical factors such as age group, American Joint Committee on Cancer (AJCC) stage, completion of chemotherapy as planned, and receipt of neoadjuvant chemotherapy showed a consistent ketorolac survival benefit in each stratum. The survival benefit of ketorolac was also evident in the proportional hazards analysis when adjusted for age at diagnosis, AJCC stage, completion of chemotherapy as planned and receipt of neoadjuvant chemotherapy. The adjusted HR for ovarian cancer–specific mortality associated with peri‐operative ketorolac (yes versus no) was 0.30 (95% confidence interval (CI), 0.11–0.88). While these findings must be interpreted cautiously because they are only partially controlled for the propensity to receive ketorolac, they suggest that, similar to the breast cancer data, peri‐operative ketorolac reduces ovarian cancer‐specific mortality.’

### How to test the ketorolac prevention of early postoperative relapses?

A chance meeting with some Nigerian physicians and scientists at a conference led to three trips to Nigeria and intense collaboration. Our main contact is co-author Prof. Osaro Erhabor, who is on Faculty of Medical Laboratory Science, Usmanu Danfodiyo University, Sokoto, Nigeria. Retsky spoke a number of times on the breast cancer perioperative NSAID project and why it might be very effective at low cost in sub-Saharan Africa where early onset (mid age 40s) and triple negative is typical. We also learned about the many potential and real problems that would be encountered doing a clinical trial in Nigeria.

Women in Nigeria typically avoid mastectomy since it is well known to them that the surgery ‘provokes’ the cancer to recur and results in death shortly thereafter. Instead they go to an herbalist for soothing ointments and then go back to their village where they die from untreated breast cancer. There is an unmet need, especially in Nigeria (190 million population) for a means to prevent surgery induced relapse that we are attempting to resolve.

After extensive discussions with our Nigerian, European and US colleagues, it was determined that all these problems are serious but solvable and that a trial could be conducted in Nigeria. Several grant proposals were submitted but rejected.

There were several significant resolutions of potential problems. For one, we had a meeting on email about possible use of mammography to detect breast cancer. Our colleague Michael Baum is a world authority on the benefits and harms from mammography [29]. He wrote the first chapter in the Springer Nature book that discusses the history of breast cancer. Baum argued that in order to use mammography, Nigeria would have to train a generation of radiologists, mammography operators and technicians to calibrate the devices that would be highly expensive and take much time. Other solutions are possible. He suggested downstaging as has been done successfully in Malaysia and India. After examining published results from Malaysia [77, 78], we agreed that downstaging would be a very reasonable method of finding breast cancer in a state treatable with perioperative NSAID ketorolac. Our Nigerian colleagues agreed.

Malaysia was able to reduce locally advanced breast cancer at presentation from 60% to 35% in 5 years with an expenditure of $35,000. They trained several hundred staff to talk to groups at churches and other gatherings. They would tell women that they should contact their staff if a breast lump was detected. They would send a nurse to determine if the lump was a cyst or possibly breast cancer. If we were to do this, and the lump was suspicious for breast cancer, at no cost the person would be taken to our facility for a diagnostic mammogram (not a screening mammogram) for confirmation. If breast cancer was confirmed and the situation was appropriate, a lumpectomy (not a mastectomy) would be conducted and intra-operative radiation therapy would be conducted. Jayant S. Vaidya, one of our colleagues originated this technology. There is potential benefit. (See chapter 11 by Vaidya in the Springer book.) Among other things, that intervention would minimise the travel for patients which would be very important in a country the size of Nigeria with few treatment centres.

We would need to provide a stand-alone facility in Abuja, Nigeria with reliable power and clean water where these interventions can be done. The total cost for all equipment and resources is under $2 million.

Chapter 6 in the Springer-Nature book was written by our Nigerian colleagues. They openly listed all the potential and real problems that would be encountered in a clinical trial in Nigeria.

We concluded that a clinical trial in Nigeria would be possible and would be *an opportunity* for the Western world to learn a simpler, far less expensive and more effective way to treat TNBC. All would benefit. However, after considering the legitimate concerns about liability from conducting a clinical trial in Nigeria, we came up with a better plan. As the result of a series of meetings with Dr. Ted James, Chief of Breast Cancer Surgical Oncology at Beth Israel Deaconess Medical Center (Harvard), we now propose to conduct a clinical trial of TNBC at Beth Israel Deaconess Medical Center under control of Ted James.

If this trial works as well as we expect, we would transfer the technology to Nigeria and provide all necessary equipment and resources to pursue the therapy there. We have agreement on this strategy from our Nigerian colleagues.

There is an unmet need in sub-Saharan Africa and particularly Nigeria with population of 190 million. The purpose of this paper is to document this situation that seems to be solvable with perioperative ketorolac.

What about postoperative bleeding with use of perioperative NSAID? An important known adverse effect from perioperative ketorolac or any NSAID is increased post-op bleeding. Others include renal dysfunction, that may be prevented by the avoidance in patients with preexisting renal insufficiency, and gastrointestinal ulceration, typically not seen with short term administration (and eventually prevented by anti-acid medications in case of long terms needs). Orthopedic surgeons have used tranexamic acid (TXA) for years to prevent post-op bleeding (A Knopf, MD). A clinical trial of TXA in mammoplasty showed 39% reduced bleeding and no other adverse effects [72]. Moreover, the Belgian randomised controlled trial did not identify any serious bleeding linked to the intraoperative use of ketorolac.

### Retrospective and prospective studies of perioperative ketorolac to prevent early relapses in breast cancer

As of this time, there have been two retrospective studies and one prospective study of perioperative NSAID ketorolac. The Forget *et al* retrospective study was impressively verified in another retrospective study also from Belgium (JNCI 2019 Desmedt, Demicheli *et al* and accompanying editorial by Ben-Eliahu *et al*) [79, 80].

A recent, modest sized, randomised controlled trial, testing a single administration of 30 mg of ketorolac, versus placebo, in patients undergoing breast cancer surgery apparently did not confirm retrospective findings [94]. Besides a possible under-powering and quite short follow-up of the trial, patients were randomised according to a not soundly standardised factor, which could have introduced confounding unbalance between the trial arms of the frequency of usual prognostic factors (e.g., TNBC, treatment modalities, BMI etc.). The possible gains from perioperative ketorolac may be so clinically rich that the question deserves a well planned and carried out randomised clinical trial leaving no queries.

The early results are summarised in a blog published online by Ralph Moss [81]. After careful analysis of the prospective trial by Forget *et al*, additional details will be published while abbreviated summaries are presented in this current document.

### The black swan metaphor

The black swan metaphor provided an appropriate way to present these data, challenge the existing treatment and explore new ideas. We have shown that there is a serious weakness in the cancer treatment paradigm and a new paradigm is needed. We propose that exploring the perioperative window will be a good place to start this process [80, 81]. Other relevant papers have been published [82–99].

## Conclusions

1. Bimodal relapse patterns are identified in breast and other cancers.
2. The unexpected observations of bimodal relapse patterns are not explainable with the current cancer paradigm that has guided cancer therapy and early detection for many years. Using computer simulation and input from a number of medical specialties, we came to the conclusion that the surgery to remove the primary tumour produces systemic inflammation for a week after surgery. This systemic inflammation apparently caused exits of cancer cells and micrometastases from dormant states and resulted in relapses in the first 3 years post-surgery.
3. Retrospective data suggest perioperative NSAID ketorolac reduces early relapses 5-fold. This still needs to be confirmed. Perhaps more than a single administration may be helpful (Forget *et al*, 2019). This may reduce breast cancer mortality by 25% to 50% at low cost and toxicity. (There are mixed reports of excessive bleeding but that is known to be reduced by 39% with Tranexamic acid [2018] and Forget *et al* 2019 data are reassuring.)
4. These data suggest transient systemic inflammation is the precipitating factor resulting in angiogenesis and single cell growth from dormancy.
5. Breast cancer runs its course in over a decade but most of the events leading to relapse seem to occur in the week after surgery.
6. This suggests metastatic progression is amplified 100-fold during the week post primary surgery.
7. After two retrospective studies, extensive clinical data analysis, and three animal models (Krall *et al* [70], Bonnelykke-Behrndtz *et al* chapter-8 [4], and Panigrahy *et al* [81]), we suggest it is time to conduct one or more prospective clinical trials. A trial is in planning stage at Beth Israel Deaconess Medical Center. This may permit us to resolve an unmet need in sub-Saharan Africa.
8. What about the cost? Drugs cost $5 per patient and there are 2 million new breast cancers per year in the world.
9. These surgery-induced effects are apparently host responses to surgery with unknown relationship with the cancer cell genotype and definitely not the fault of the surgeons.
10. This seems to be a general effect not just limited to breast cancer.

We are analysing these data and are now proposing to test the perioperative NSAID at Beth Israel Deaconess Medical Center with Triple Negative Breast Cancer patients, the category that could respond best to the perioperative NSAID. If this works as well as we expect, we would then transfer this technology to Nigeria where an early onset type of TNBC is common. Women in Nigeria typically avoid mastectomy since it is well known to them that the surgery ‘provokes’ the cancer to recur and results in death shortly thereafter. Instead they go to an herbalist for soothing ointments and then go back to their village where they die from untreated breast cancer. There is an unmet need especially in Nigeria (190 million population) for a means to prevent surgery induced relapse that we are attempting to resolve.

## Funding declaration

This project was supported by grant 1R43CA65314 from NIH (PI: M Retsky) and 100484 from The Komen Foundation (PI: Rick Rogers).

## Conflicts of interest

M Retsky has three patents pending. No other conflicts of interest are reported.

## Figures and Tables

**Figure 10.**
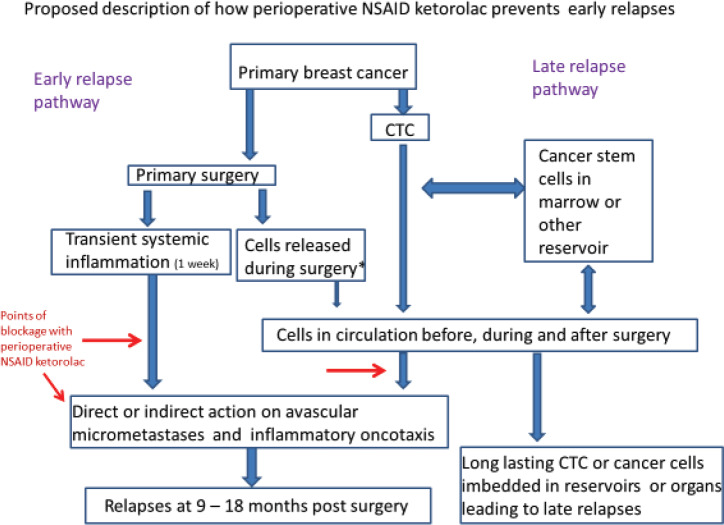
Proposed mechanism along with the conventional mechanisms.

**Figure 11.**
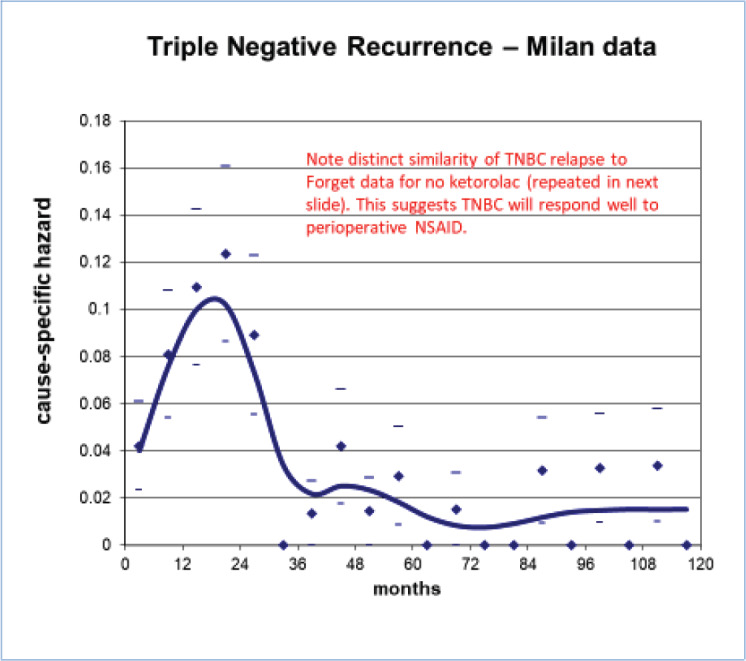
TNBC recurrence data from Milan as seen in Figure 11 is quite similar to the no-ketorolac arm of Forget *et al* data (Figure 7). This strongly suggested to us that perioperative ketorolac will be effective in sub-Saharan Africa where TNBC is common (See chapter 6.) The low cost will be another enhancing factor. Low and Middle Income Countries have 70% of the world’s cancer burden but 5% of the financial resources [75].

**Figure 12.**
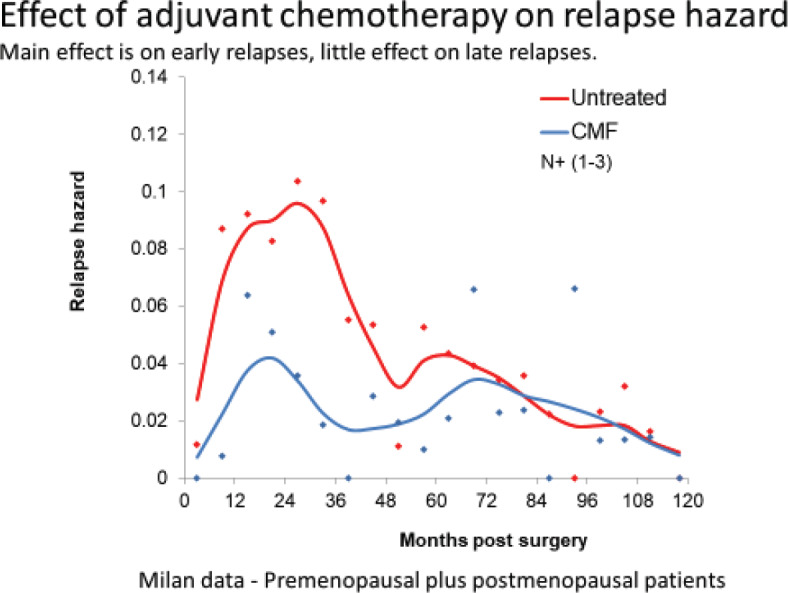
Data from Milan show that the benefit of adjuvant chemotherapy is mainly to reduce the early relapses. This is consistent with the analysis based on the computer simulation.

**Table 1.** Findings and interpretations regarding peaks of relapses after breast cancer surgery.

	Finding	Interpretation
Results of computer simulation	Early (dominant) peak composed of two previously unreported surgery-induced relapse modes at 10 months and at 30 months.	Concordant with the clinical observations.
10-month peak	20% of premenopausal node-positive patients undergo this relapse mode. It is 5:1 node-positive to node-negative and 2:1 pre- compared to postmenopausal.	Avascular micrometastases induced to vascularise and relapse appears at 10 months.
30-month peak	Together the two surgery induced relapse modes comprise 50% to 80% of relapses (increasing with tumour size and nodes positive).	Previously inactive single cells are induced to divide and then stochastically vascularise with relapse peak at 30 months.
Late shallow peak (50–200 months)	The ‘natural history’ of breast cancer.	The benefit of surgery to reduce metastases first appears at 5 years.

**Table 2.** Similar bimodal relapse pattern seen in other solid cancers.

Cancer type	First author, year of publication	Reference
Pancreatic	Deylgat *et al*, 2011	[12]
Melanoma	Tseng *et al*, 2009	[13]
	Demicheli *et al*, 2014	[14]
Non-small cell lung cancer	Demicheli *et al*, 2012	[15]
	Kelsey *et al*, 2013	[16]
	Maniwa *et al*, 1998	[17]
Prostate	Hanin *et al*, 2011	[18]
	Weckermann *et al*, 2009	[19]
Osteosarcoma	Smithers, 1968	[6]
	Tsunemi *et al*, 2003	[20]
Esophageal	Zhu *et al*, 2015	[21]
Head and neck	Lama *et al*, 2011	[22]
Nasopharyngeal	Xia *et al*, 2013	[23]
Testicular	Lange *et al*, 1980	[24]
Colorectal	Schack *et al*, 2019	[25]
Ovarian	Guo *et al*, 2015	[26]
Glioma	Hamard, 2016	[27]
	Ratel, 2016	[28]

**Table 3.** Comments from Celsus and Galen on treating breast cancer 2000 years ago.

**Aulus Cornelius Celsus** (30 BC–38 AD)
First there is the cacoethes, then carcinoma without ulceration, then the fungating ulcer. None of these can be removed but the cacoethes: the rest are irritated by every method of cure. The more violent the operations the more angry they grow. After excision it recurs, bringing with it the cause of death, whereas at the same time by using no extirpation protract lives, notwithstanding the disorder, to an extreme old age.
**Galen of Pergamum** (131–203 AD)
We have often cured this disease in the early stages, but after it has grown to a noticeable size no one has cured it with surgery.
**Interpretation**
Both Celsus and Galen knew about the stimulation of distant metastases after breast tumour removal especially if the tumour was more advanced than ‘cacotheses’. But with cacotheses, a patient could be cured with tumour removal even without benefit of pain or infection control. That was equally remarkable.

**Table 4.** Survey of observations and hypotheses among the fields of surgery, dormancy, inflammation, circulating tumour cells, wounding and immunology.

Hypothesis or observation	Reference
‘the perioperative period can be considered a “perfect storm” of immunosuppression and inflammation in the presence of residual or circulating tumour cells.’	[48]
Genetic damage lights the fire and inflammation is the fuel that feeds the flame of cancer	[49]
Dormancy is a well accepted phenomenon in cancer	
Surgery results in systemic Inflammation for a week (colon and breast) IL-6 in serum	[50, 51]
Neutrophils are generated in large numbers after injury	[52, 53]
Neutrophils can extravasate and provide Extracellular Traps to capture cancer cells	[52, 53]
Localisation of secondary tumours at points of injury (1914 report) (Jones and Rous)	[54]
Description of cancer as similar to wound healing (Chaffer, Dvorak)	[56, 57]
Perioperative ketorolac reduces use of opioids (proangiogenic).	[58]
Bimodal relapse patterns apparent in solid tumours	[current paper]
Tumour grows at any site of wounding in Rous sarcoma avian model, controlled with inflammation	[43,44]
Daily aspirin can lower mortality of breast and colon cancer	[59]
Platelets sequester angiogenesis regulators (Klement)	[62]
Cancer patients have circulating tumour cells that correlates with prognosis particularly in TNBC (Karhade)	[71]
Peak in CTC after mastectomy but 3–7 days later (Camara)	[65]
Localised metastatic disease at site of recent physical trauma - termed ‘Inflammatory oncotaxis’ (Walter)	[55]
Capillary permeability increased from 30 to 70 kDa to 2,000 kDa after inflammation. (Egawa)	[67]
The IL-6 serum level correlates with prognosis in many cancers (Lippitz)	[69]
